# A randomised controlled trial: can acupuncture reduce drug requirement during analgosedation with propofol and alfentanil for colonoscopy? A study protocol

**DOI:** 10.1186/s12906-015-0936-5

**Published:** 2015-11-16

**Authors:** Susanne Eberl, Nelson Monteiro de Olivera, Benedikt Preckel, Konrad Streitberger, Paul Fockens, Markus W. Hollmann

**Affiliations:** Department of Anaesthesiology, Academic Medical Centre, University of Amsterdam, Meibergdreef 9, Amsterdam, 1100 DD The Netherlands; Department of Anaesthesiology and Pain Therapy, Inselspital, Bern, Switzerland; Department of Gastroenterology & Hepatology, Academic Medical Centre, University of Amsterdam, Meibergdreef 9, Amsterdam, 1100 DD The Netherlands

**Keywords:** Procedural sedation, Propofol, Acupuncture, Colonoscopy

## Abstract

**Background:**

The number of colonoscopies tremendously increased in recent years and will further rise in the near future. Because of patients’ growing expectation on comfort during medical procedures, it is not surprising that the demand for sedation also expands. Propofol in combination with alfentanil is known to provide excellent analgosedation, however, its use is associated with respiratory and cardiovascular depression. Acupuncture could be a technique to reduce drug requirement while providing the same level of sedation and analgesia.

**Methods/design:**

The study will be performed as a single centre, randomised, placebo controlled trial. 153 patients scheduled for propofol/alfentanil sedation during colonoscopy will be randomly assigned to receive electroacupuncture (P6, ST36, LI4), sham acupuncture, or placebo acupuncture. Following endoscopy patients and gastroenterologists have to fill in questionnaires about their sedation experiences. Additionally, patients have to accomplish the Trieger test before and after the procedure. Patient monitoring includes time adapted HR, SpO_2_, ECG, NIBP, exCO_2,_ OAA/S, and the Aldrete score. The primary outcome parameter is the dosage of propofol necessary for an adequate level of sedation to tolerate the procedure (OAA/S < 4). Effectiveness of sedation, classified by satisfaction levels measured by questionnaires is the secondary outcome parameter.

**Discussion:**

Moderate to deep sedation using propofol is increasingly applied during colonoscopies with a high satisfaction level among patients despite well-known hemodynamic and respiratory side effects of this hypnotic agent. Acupuncture is known to attenuate gastrointestinal discomfort and pain. We hypothesize that the combination of conventional sedation techniques with acupuncture may result in equally satisfied patients with a lower risk of respiratory and hemodynamic events during colonoscopies.

**Trial registration:**

This trial is registered in the Nederland’s Trial Register NTR 4325.

The first patient was randomized on 13 February 2014.

## Background

Colonoscopies are known to be uncomfortable for patients. Pain and vasovagal reactions often necessitate administration of sedative or analgesic agents, or even the combination of both.

Propofol as a sedative agent has a rapid onset and termination of action, making it the ideal choice for endoscopic procedures. The most important disadvantage -especially in combination with an opioid- is the risk of a rapid change from conscious to deep sedation or even general anaesthesia with consecutive cardiorespiratory depression. Therefore, an important clinical question is, how to reduce the necessary dosage of propofol and the risks involved while retaining a satisfactory sedation for patient and endoscopist. Additional acupuncture could be an option to solve this problem.

Acupuncture is used worldwide for different indications, mainly for pain management [1–4]. However, only few publications exist with respect to the role of acupuncture for sedation during gastrointestinal procedures. Fanti et al. could show that the combination of LI4, S36, SP6 and SP9 can decrease the demand for sedative drugs [5]. Leung et al. [6] demonstrated that electro-acupuncture at P6, ST36 and LI4 can reduce discomfort and pain during barostat-induced rectal distension. Ni et al. [7] found a significant reduction in stress response and dosage of midazolam during colonoscopy by acupuncture using ST36, ST37, SP6, SP9, and LI4.

However, it is not clear whether these effects are a result of acupuncture or simply a strong placebo effect [8]. The theoretical basis of acupuncture is still not known. Two reviews report that there is no evidence for an effect of ‘true’ acupuncture over acupuncture at “non points” (sham-acupuncture) [9, 10]. Release of neurotransmitters, parasympathetic disinhibition, or even placebo effect could also be explanations for the acupuncture results. Until now no study demonstrated a link between these effects and specific acupuncture points.

## Methods/design

### Aim of the study

We hypothesize that “true” acupuncture causes besides an analgesic also a sedative effect that will lead to a reduction of the dosage of propofol needed for satisfactory sedation during colonoscopies.

Satisfactory sedation is defined as an Observer’s Assessment of Alertness/Sedation Scale (OAA/S) level 2/3. Target controlled infusion (TCI) of propofol is adapted to achieve this targeted level of sedation.

To test this hypothesis we will compare three groups. Group 1 will receive verum-acupuncture at the traditional acupuncture points P6, ST36, LI4, group 2 will get sham-acupuncture and group 3 will be the control group with placebo needles.

All three groups will receive standard sedation with propofol TCI/alfentanil.

Primary endpoint of the study is the used dosage of propofol - in combination with a limited dosage of alfentanil.

Secondary endpoint is patients’ and gastroenterologists’ satisfaction with the procedure, determined with validated questionnaires.

### Trial design

The study is designed as a single centre, randomised, placebo controlled trial and reported following the STRICTA (Standards for Reporting Interventions in Clinical Trials of Acupuncture) reporting guidelines [11], and the CONSORT statement [12].

### Participants

#### Number of patients’ needed

Sample size calculation is based on observational propofol data from previous colonoscopies, collected in our hospital sedation database. Primary end-point is the difference in the dosage of propofol. The mean dosage of propofol during colonoscopy is 441 mg, with a standard deviation of 176 mg [13]. We will need to study 47 subjects in each group, given a power of 0.80 and a type I error of 0.05 to reduce propofol requirement by about 25 %. Considering a dropout rate of 10 %, the estimated sample size will be 51 patients per group, thus a total of 153 patients will be randomized.

#### Eligibility

The study takes place at the Department of Gastroenterology and Hepatology in the Academic Medical Centre (AMC) of the University of Amsterdam beginning February 2014 to August 2016. Eligible patients for participation in this clinical trial are those planned to undergo elective diagnostic or therapeutic colonoscopy under propofol/alfentanil sedation, aged above 18 years, and ASA classification I-III, who give written informed consent.

#### Exclusion criteria

- Age < 18 years
- ASA classification VI and V
- Allergic reaction to planned medication in the patients’ medical history
- Nickel allergy
- Pacemaker/Implanted Cardioverter Device
- Psychiatric and neurologic disorders
- Use of anticoagulants
- Refusing sedation

The number of excluded patients and the reasons for their exclusion will be reported according to the CONSORT statement.

#### Consent

Patients scheduled for an elective colonoscopy will be asked – after checking inclusion and exclusion criteria - by phone to participate. If they are eligible for inclusion and interested in participating in the study, patient information will be sent. Final inclusion occurs after written informed consent. If they deny taking part they will be sedated with propofol and alfentanil without any form of needling. Cardiovascular monitoring using oxygen saturation (SpO_2_), heart rate (HR), ECG, non-invasive blood pressure (NIBP) and exhaled CO_2_ (exCO_2_) will be performed in all patients at 5-min intervals. The investigator (physician performing the acupuncture or physician performing the examination) can decide to withdraw a subject from the study for urgent medical reasons (allergic reactions, extreme pain on acupuncture points, acute health problems).

### Randomization

After the patient has signed the informed consent form, randomization will be performed as a permuted block randomization (RITA version 1.31). Allocation to treatment group will be performed by sealed consecutively numbered envelopes.

One experienced and qualified staff acupuncturist (4 years in practice) will perform the intervention, but is not further involved in the study. After the patient is sedated and before colonoscopy starts the acupuncturist will open the allocation envelope.

### Intervention

After randomization patients are prepared for endoscopy. Glycopyrrolate 0.2 mg and lidocaine 50 mg are administered intravenously, and patients are positioned in right lateral position. Via a nasal cannula 2 L/min of oxygen will be given from start of sedation till the end of the endoscopic procedure.

Each group will receive sedation - supplied by a specialized anaesthesia nurse (sedation specialist) - using a propofol TCI system that is weight and age adapted. The Marsh pharmacokinetic model is employed to attain a pre-set propofol plasma target level.

The targeted sedation score – measured by Observer’s Assessment of Alertness/Sedation OAAS Scale - should be 2 or 3, which means patients response maximal lethargic if their name is called loudly and/or repeatedly or after mild prodding or shaking.

If OAA/S ≥ 4, which means that the patient is too alert or agitated to tolerate the procedure, additional sedation will be provided with step up of the TCI.

Additionally, when starting propofol TCI, patients will be given 1.5 μg/kg alfentanil.

We use the Behavioural Pain Scale for not-intubated patients (BPS-NI) [14]. The BPS–NI evaluates three behavioural domains (i.e., facial expression, movements of upper limbs and vocalization). Each domain contains four descriptors that are rated on a 1–4 scale, with a value ranges from 3 (no pain) to 12 (most pain). Patients with BPS-NI ≥ 7 were given an additional dose of 1,5 μg/kg alfentanil.

**Fig. 1:**
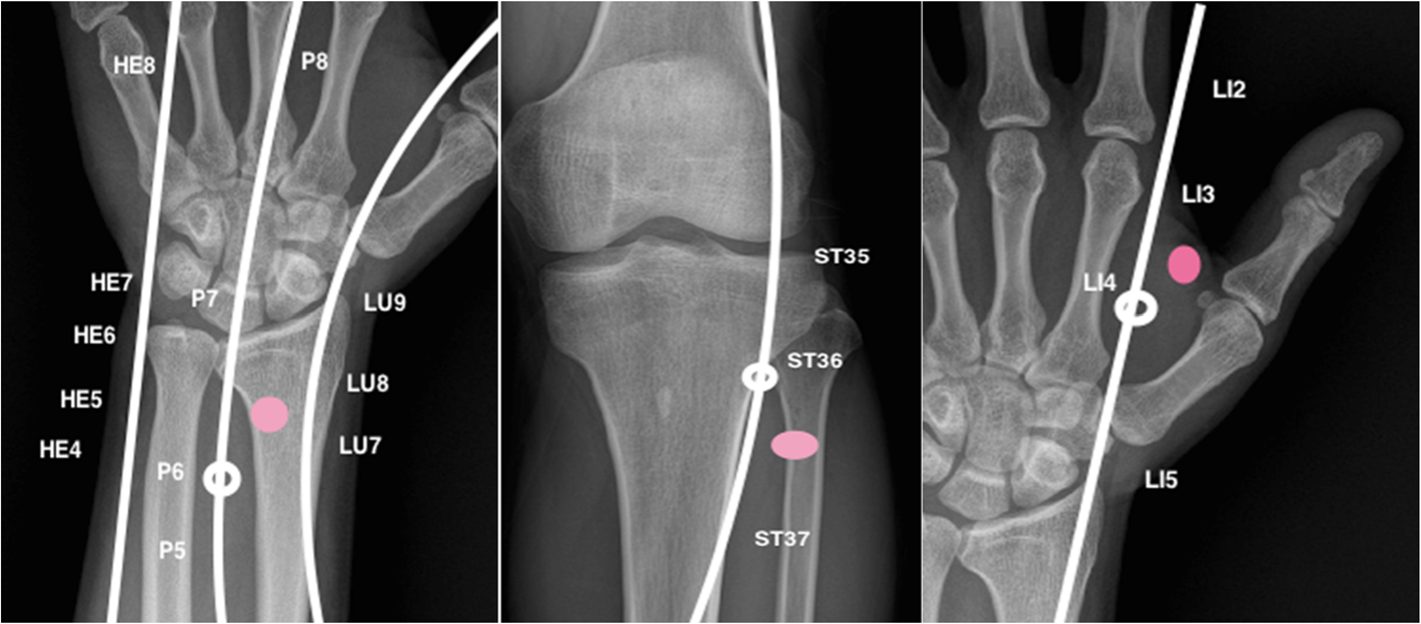
Acu-points (white) vs. sham-acupoints (red)

After starting propofol sedation and reaching the targeted sedation score (OAAS 2/3), the needles are placed by the acupuncturist according to allocation (Fig. 1) and connected to a six-channel programmable electrical stimulator (SVESA 1031, Germany, Munich). Stimulation frequency (1–10 Hz) should be high enough to provoke muscle contraction. In the control group, the stimulator will likewise be connected but without electrical stimulation. The intervention with electroacupuncture will be stopped as soon as the colonoscopy is finished.

After endoscopic examination the position of the needles will be checked. If some of the needles are not in place any more after colonoscopy, it will be documented in the CRF.

Group 1 will have verum acupuncture unilateral on three points according the theory of Traditional Chinese medicine. Nickel needles (Asia-med, Germany, Special No. 16 (0.30x30 mm / (Gauge 8x1.2") will be placed and left in their position for five minutes prior and throughout the endoscopic procedure. We choose the following points, which are considered relevant both - for sedation and for abdominal distension: Pericardium 6 (P6), Stomach 36 (ST36), and Large intestine 4 (LI4). The localization of these points is described in units of cun. Cun is a traditional Chinese unit of length. One cun is defined as the width of a persons thumb, whereas four fingers are defined as 3 cun. This corresponds to about 3 cm of length.

There are no specific acupuncture points known for their sedative effect. Large intestine 4 (LI4), Pericardium 6 (P6) and Stomach 36 (ST36) is the most used combination to reduce gastrointestinal discomfort and pain [5, 7] with a consecutive sedation side effect [15].

- Pericard 6 (P6) is stimulated with various acupuncture techniques, especially used for preventing and treatment of nausea and vomiting. It is located on the skin of the anterior forearm, two cun above to the wrist crease, between the tendons of m. palmaris longus and m. flexor carpi radialis.
- The second point is Stomach 36 (ST36). It is located below the knee, one-finger breadth lateral to the anterior crest of the tibia and 3 cun inferior to the hollow that is formed below the patella, and lateral to the patellar ligament when the knee is flexed. It is supposed to be the acupoint with the most influence on the gastro-intestinal tract.
- These two acupoints are combined with Large intestine 4 (LI4) located on the dorsum of the hand between the first and second metacarpal bones at the midpoint of the second metacarpal bone, and close to its radial border. LI4 is one of the most used acupoints, frequently in combination with other acupoints for the treatment of pain and intestinal diseases [16].

Group 2 will receive Sham-acupuncture with the same sort of needles. The needles will be placed 1 cm distal and lateral the used acupoints (P6, ST36, LI4) on non-points - locations that are not known as acupuncture points.

Group 3 will have sham acupuncture with placebo needles (Streitberger needles, Asia-med, Germany, 0.30x30 mm / (Gauge 8x1.2") on the same points used for sham- acupuncture to exclude a significant effect of acupressure on the regular acupuncture points.

The Streitberger needle looks like a real needle but has a blunt tip whose shaft telescopes into the handle during application without penetrating the skin [17].

Resident and senior endoscopists – reflecting the spectrum of a teaching hospital-will perform colonoscopy.

A not blinded medical student will document and monitor the procedure in the endoscopy and recovery room.

The other attending parties (patient, sedation anaesthesia nurse, endoscopist, endoscopic nurse) are blinded and not informed about the allocation.

At arrival in the recovery room patients will be monitored by SpO_2_, ECG and NIBP only.

The level of recovery from sedation and the return of psychomotor fitness will be assessed using the modified Aldrete Score directly at the end of the procedure, at arrival in the recovery room, 30 and 60 min thereafter. All patients will stay in the recovery room for at least 60 min. Ready for discharge (virtual discharge) will be declared when an Aldrete Score ≥9 or pre-procedure score is met.

“Discharge criteria” require further that the patient is awake and alert with stable vital signs, is able to ambulate without assistance, and is free of side effects of the drugs employed during the procedure.

### Primary objective

#### Definition of primary endpoint

The primary aim of this randomised controlled study is to determine whether application of acupuncture decreases the total dosage of propofol needed to reach a sedation level of OAAS 2/3 during colonoscopies.

We also want to clarify, whether a reduction in the dosage of sedatives will result in a reduction of side effects of sedation, especially respiratory and circulatory depression, nausea and vomiting, and may lead to a faster recovery.

#### Assessment of primary endpoint

We will record total amount of propofol and alfentanil, and all other drugs administered, psychomotoric recovery, hemodynamic and respiratory parameters and events, and all actions visibly taken to prevent or treat these problems, such as chin lift/jaw thrust, stimulating the patient, temporary mask ventilation, use of ephedrine or phenylephrine, etc.

Psychomotoric recovery is assessed by the Trieger test [18]. For this test patients have to connect points forming a figure with a line using a pen, before and 30 min after the procedure. Missing points as well as the distances of the line from the true points are noted and rated as score post/preprocedural.

HR, NIBD and SpO2 are reported as area under the curve (AUC). Surrogate parameters of pulmonary and cardiovascular events are a decline in SpO_2_ to less than 90 %, or breathing frequency less than 6 breath/min; a change in HR to less or more than 20 % of baseline or occurrence of any arrhythmia’s; NIBP less or more than 20 % of the first value determined. The events will be recorded, if they are longer lasting than 5 min of request intervention.

### Secondary objective

#### Definition of secondary endpoint

Patients’ and endoscopists’ satisfaction with the entire procedure is defined as secondary endpoint.

#### Assessment of secondary endpoint

Satisfaction will be evaluated by means of validated questionnaires [19].

Before the procedure, patients and endoscopists will fill in a questionnaire concerning basic information and specific expectations of the procedure. Following the procedure and before discharge, patients and gastroenterologists are urged to fill in questionnaires modified from the Patient Satisfaction with Sedation Instrument (PSSI), respectively Clinical Satisfaction with Sedation Instrument (CSSI). A follow-up telephone call will be made 24 h later asking questions from part two of the PSSI questionnaire concerning global satisfaction.

Vargo et al. [19] developed these questionnaires with finally 4 subscores to describe patients and 3 subscores to describe endoscopists’ satisfaction. Subscores for the PSSI contain questions about sedation delivery, procedural recall, sedation side effects, and global satisfaction. Subscores for the CSSI refer to corresponding issues among gastroenterologists: Sedation administration, recovery/ post-op and global satisfaction. Patients and endoscopists could classify their satisfaction or dissatisfaction during the procedure ranging from 7 = very satisfied to 1 = very dissatisfied.

The dosage of alfentanil as objective parameter of suffered pain during procedure will also be measured.

### Statistical analysis

Statistical analyses will be performed using SPSS statistics.

All data will be checked for normal distribution using the Kolmogorov test. For normal distributed, continuous variables an independent Student’s t - test will be used and the variables will be presented as mean ± SD. For normal distributed categorical variables Pearson chi square test will be applied and variables will be allegorized as number and/or percentage of total. Not normally distributed data will be compared using multifactorial ANOVA (Kruska Wallis) or the Whitney *U*-test where appropriate. These variables will be presented as median/25/75 percentile. A *p* -value < 0.05 will be considered statistically significant. Confidence intervals are mentioned where appropriate.

### Ethical approval

This trial will be conducted in accordance with the protocol and in compliance with the moral, ethical, and scientific principles governing clinical research as set out in the Declaration of Helsinki (1989) and Good Clinical Practice (GCP). The Departments of Anesthesiology and Gastroenterology & Hepatology of the Academic Medical Centre of Amsterdam are responsible for the design and conduct of the trial. The protocol was designed according to the Standards for reporting Interventions in Clinical Trials Acupuncture (STRICTA) [11]. It is registered in the Nederland’s Trial Register (NTR NTR4325). Ethical approval was obtained from the Medical Ethics Committee of the Academic Medical Centre, Amsterdam, the Netherland (NL). The National Authority, the Central Committee on Research Involving Human Subjects (CCMO), performed a marginal review and there were no objections to perform this study (NL36861.018.11).

## Discussion

One might discuss to analyse the data using a non-inferiority approach because sedation quality after application of acupuncture should be at least as good as using medication only.

However, our motivation in the end to choose for the superiority approach was: During this trial acupuncture is performed by only one experienced acupuncturist. Implementing acupuncture in our daily anaesthesia sedation praxis would mean additional engagement of anaesthesia personal to learn and perform acupuncture. This investment will only be cost-effective if the dosage of propofol is reduced or - in case of the dosage of propofol is not influenced, there is an improvement of satisfaction among patients and endoscopist.

Alongside, safety concerns of deep sedation performed with propofol still remain. Cote et al. showed that hypoxemia occurred in 12.8 % of 799 patients sedated for endoscopic procedures with propofol applied by trained anaesthesia nurses [20]. In 14.4 % of the patients airway manoeuvres were necessary to prevent hypoxemia. In our own previous study looking at 180 patients undergoing colonoscopy, 47 % of all patients with propofol sedation experienced at least one respiratory incident and 87 % had at least one hypotensive event [13]. Minimizing those propofol related risks by means of a superior sedation form is therefore an important goal to make sedation procedures safer.

Aside these considerations all groups receive propofol sedation and therefore using placebo needles is not an unethical treatment.

Another point of discussion comes up with the question: Does acupuncture really have a sedative effect or is this effect the consequence of its analgesic properties.

Fanti et al. studied three groups of patients scheduled for colonoscopy [5]. Group 1 got acupuncture, group 2 sham-acupuncture and group 3 placebo. All groups were premedicated with 0.02-mg/kg midazolam before, and no analgesic agent was used. If patients complained about pain during procedure they received additional boluses of midazolam. To medicate a patient moaning about pain with a sedative and to conclude that acupuncture can decrease the demand for sedatives during colonoscopy is not the correct conclusion.

So the question whether acupuncture can reduce the dosage of sedative, and be implemented into the clinical routine as a complementary sedative method has still to be answered [21].

## Trial status

The first patient was included at 13 February 2014. We consider including 3 patients per week.

## Abbreviations

CSSI: Clinical satisfaction with sedation instrument
exCO2: Exhaled CO2
GCP: Good clinical practice
HR: Heart rate
NIBP: Non-invasive blood pressure
NRS: Numeric rating score
OAA/S: Observer’s assessment of alertness/sedation
PSSI: Patient satisfaction with sedation instrument
SO_2_: Oxygen saturation
STRICTA: Standards for reporting interventions in clinical trials of acupuncture
TCI: Target controlled infusion
